# Factors influencing the intention to use the ICD-11 among medical record officers (MROs) and assistant medical record officers (AMROs) in Ministry of Health, Malaysia

**DOI:** 10.1038/s41598-024-60439-2

**Published:** 2024-04-30

**Authors:** Erwyn Chin Wei Ooi, Zaleha Md Isa, Mohd Rizal Abdul Manaf, Ahmad Soufi Ahmad Fuad, Azman Ahmad, Mimi Nurakmal Mustapa, Nuraidah Mohd Marzuki

**Affiliations:** 1 Department of Public Health Medicine, Faculty of Medicine, National University of Malaysia, Kuala Lumpur, Malaysia; 2 Health Informatics Centre, Planning Division, Ministry of Health Malaysia, Putrajaya, Malaysia

**Keywords:** Health care economics, Health policy, Health services

## Abstract

The transition of ICD has never been a straightforward initiative. As nations transition to ICD-11, ensuring its acceptance among the users is essential. To our knowledge, there are limited studies about the instrument and ICD-11 adoption. Therefore, the purpose of this study was to design an instrument and investigate the factors influencing the intention to use the ICD-11 among medical record officers (MROs) and assistant medical record officers (AMROs) at Ministry of Health (MOH) Malaysia facilities. Based on the current literature, a model based on the decomposed theory of planned behaviour (DTPB) was proposed. The model consisted of 13 dimensions and 12 hypotheses identified from previous studies. Using PLS-SEM, 185 survey data points were analysed. The study findings showed that ten factors have a significant impact on the suggested model. Users' subjective norm was the most influential factor in their intention to use ICD-11. Unexpectedly, perceived usefulness and was found to have no significant influence. This study is important for policymakers in strategising ICD-11 implementation efforts. This study's novelty lies in applying a DTPB theory model in the context of the intention to use ICD-11.

## Introduction

Healthcare institutions have an insatiable appetite for data and retain vast amounts of patient-level information in clinical records. However, only a small portion of these data can be used in day-to-day decision-making because of standardisation issues^1^. The International Statistical Classification of Diseases and Related Health Problems (ICD) enables the alphanumeric coding of disease diagnoses and other health problems, which allows easier data storage, retrieval, and analysis^2^. ICD has served as the primary foundation for comparing statistics on causes of death and morbidity for more than a century^3^.

The 72nd World Health Assembly adopted the 11th revision of the International Classification of Disease (ICD-11) in 2019^4^. Based on formal ontology, the ICD-11 is a distinct, adaptable, and effective health information system used in information technology (IT) infrastructures and with other classifications and terminologies^5^. Therefore, the use of ICD-11 introduces a transition from a largely manual workflow to work processes involving the electronic use of the ICD^6^. Essentially, users must learn to use the ICD-11 and adapt to new and fundamentally diverse methods of executing business processes in the healthcare setting.

There has been an increasing number of studies on the use of ICD-11. However, existing studies have focused mainly on training evaluation^7^ or from the perspective of clinicians^8^. To the best of our knowledge, limited studies have been performed on designing and applying an instrument for measuring intention to use the ICD-11. In the context of medical records personnel, which is this study's subject, studies on the topic are even more limited^7^. MROs and AMROs, also known as health information management professionals (HIMs) in developed countries, are tasked with enhancing the standards for clinical documentation to provide data and information for patient care^9^.

In developing countries such as Malaysia, MROs and AMROs are focused on nine specific areas ranging from disease and procedural codification, policies, and information management to quality assurance^10^. MROs and AMROs are essential in ensuring the quality of diagnosis documentation, ICD coding and, ultimately, the publication of the documented data at the national level. To bridge this gap, the current study focused on the scale validity of a model that incorporates variables influencing the intention to use the ICD-11 in the context of Malaysia, a developing nation. The PLS-SEM technique assessed the model and tested the structural hypotheses.

## Literature review

The decomposed theory of planned behaviour (DTPB) has demonstrated that decomposing attitudes, subjective norms, and perceived behavioural control increases the explanatory power of intentions to use technological innovations such as the ICD-11^5,11,12^. First, the theory focused on compatibility, perceived ease of use, perceived usefulness for users' attitudes, interpersonal influence, and external influence for the subjective norm and facilitating conditions and self-efficacy for perceived behavioural control. The theory then progresses from the three determinants of user behaviour (attitude, subjective norms, and perceived behavioural control), which shape users' intentions^11,13^.

### Intention to use the ICD-11 among MROs and AMROs

Pragmatically, ICD-11 implementation in Malaysia was still in an early stage at the time of data collection^14^. Thus, intention over actual usage is desirable, allowing investigation of MROs' and AMROs' acceptance at a time when more countries and health organisations were implementing the ICD-11^15^. In addition, implementing the ICD-11 at MOH facilities is a mandated effort involving all systems. In a mandatory environment, intentions are more suited than actual usage because they are measured concurrently with beliefs^16,17^. Users may hold an unfavourable opinion and not want to adopt the innovation^16,18^. Although they will eventually adopt the new coding system, they do so because there are no other options available and at the expense of the additional time and resources for the latest technology to be implemented successfully^16,19^.

Therefore, barriers to innovative technology such as the ICD-11 impact the intention and eventual usage of the ICD-11^15^. In the context of ICD transition, in previous studies, users faced significant challenges with minimal experience in the new and more complex coding system^20,21^, leading to inaccurate and unspecified coding^22,23^, which requires long and expensive training^24^. In ICD code selection, having too many options can be worse than having too few options, as this leads to increased inter- and intraobserver variability and improper use, especially if the system is time-consuming^25,26^.

### Instrument needs

In general, researchers design instruments with sufficient relevant items and constructs. The goal is to capture the key constructs to predict the behaviour of interest. Therefore, this study aimed to develop and validate a scale to model the factors influencing the intention to use the ICD-11 among MROs and AMROs at MOH facilities. Previous research has focused on developing and validating instruments on technology intentions or adoptions, which has led to the development of related theories. For example, the Technology Acceptance Model (TAM)^27^, the Unified Theory of Acceptance and Use of Technology (UTAUT)^28^ and the Theory of Planned Behavior (TPB)^29^. In various settings and contexts, these models have been modified and validated^30–32^.

The TPB is found to have a greater ability to predict and explain behaviours in a mandatory environment than the UTAUT and TAM^16,33^. However, the decomposed TPB (DTPB) model has superior predictive and explanatory power^12^. DTPB has been used extensively, involving innovations in the fields of education^34^, finance^35^ and healthcare^11,36^. Therefore, ICD-11, as a technological innovation in the context of this study^5^, we applied DTPB in a different setting, context and population involving MROs and AMROs in Malaysia.

### Model and hypotheses

**Figure 1:**
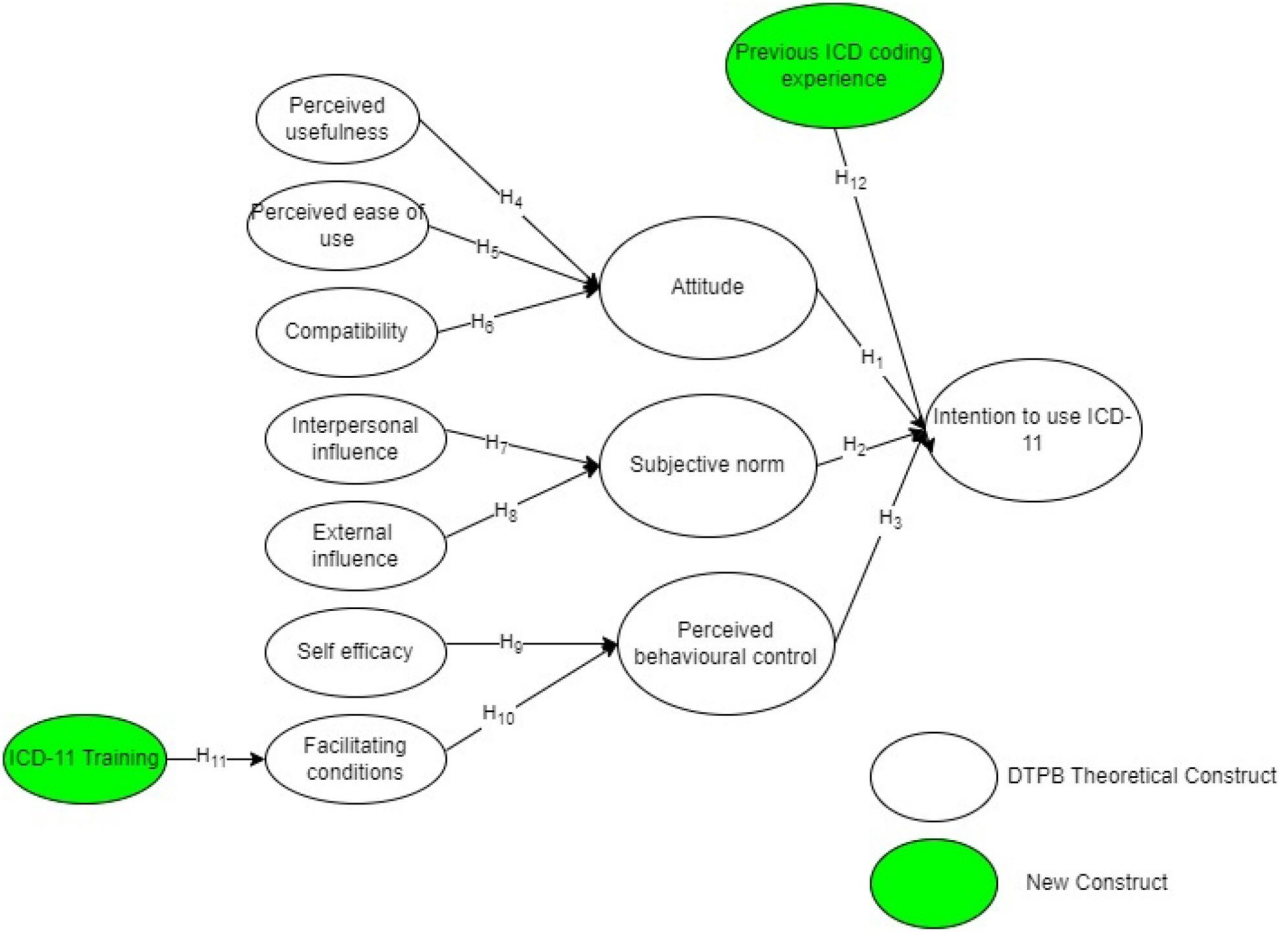
Proposed model.

For this study, we outlined a model consisting of twelve hypotheses for the structural model. Figure 1 portrays the study's proposed model. The suggested model and scale refer to the intention to adopt the ICD-11 context at the Ministry of Health facilities involving MROs and AMROs.

#### Intention to use ICD-11

This study defines intention to use ICD-11 as the readiness of the users to act in using a newly introduced innovation^37,38^. Intention to use is hypothesised to be influenced by attitude (H1), subjective norm (H2), perceived behavioural control (H3) and previous ICD coding experience (H12)^12^. In the context of this study, the intention to use is explored from the point of view of the MROs and AMROs from the MOH facilities.

#### Attitude

In this study, the attitude was defined as MROs' and AMROs' tendency to accept or reject the use of the ICD-11^39^. It has been established that attitude is an essential predictor of a person's intention to use a newly introduced technology^12,40^. In the healthcare context, a positive attitude toward the new system improves the chances of the users' intention to adopt the system^11^. For example, in the use of electronic health records among doctors^40^. Therefore, the following hypothesis is proposed.

H1: Attitudes towards ICD-11 positively influence MROs' and AMROs' intentions to use ICD-11.

#### Subjective norm

Subjective pressure from the social groups of MROs and AMROs influences the adoption intentions of ICD-11 codes. Social groups are usually people who are essential to respondents and consist of peers, colleagues, and supervisors in the workplace^12,41^. Previous studies have shown that the stronger the influence of these groups of people on the respondents, the greater the respondents' intention to perform the behaviour of interest^42^. In other words, reactions and feedback from people who are important to users will influence users' intention to use or not use the innovation. Mathai et al. reported that subjective norms positively influence intentions in the context of system use in healthcare^11^. As a result, the following hypothesis is proposed:

H2: Subjective norm positively influence MROs' and AMROs' intentions to use ICD-11.

#### Perceived behavioural control

According to Ajzen, MROs' and AMROs' perceptions of the readiness of resources and materials to use an innovation such as the ICD-11 are defined as perceived behavioural control^29^. In line with DTPB^12^, users' perceived behavioural control should influence the intention to use ICD-11. A study by Gupta et al. reported that perceived behavioural control among individuals has a positive relationship with the usage intention of metaverse in healthcare^43^. Thus, this study hypothesises that MROs and AMROs are more likely to intend to use the ICD-11 when they feel they have more control over it.

H3: Perceived behavioural control positively influences MROs' and AMROs' intentions to use the ICD-11.

#### Previous ICD coding experience

Previous ICD coding experience is defined as prior use of ICD^44,45^. Past experiences play a significant role in determining intention towards a behaviour like using an innovation^46^. This is because experience allows new information to be more relatable to memory, thereby helping in the decision to use innovations^47^. In the context of ICD, previous experience has played a significant role in the acceptance of new classification systems, and it is hypothesised that^48,49^:

H12: Previous ICD coding experience positively influences MROs' and AMROs' intentions to use the ICD-11.

### Decomposition of attitude

#### Perceived usefulness

According to the proposed model, attitudes toward the ICD-11 are determined by perceived usefulness, perceived ease of use and compatibility. A system perceived to be useful by users will positively influence intention and, subsequently, the decision to use it. Hence, in this study's context, perceived usefulness is defined as the extent to which MROs and AMROs believe that the ICD-11 can improve their performance at work^27^. Studies have shown that users' perceived usefulness of a new technology influences their attitude toward the technology^50^. For example, in a study of users of healthcare-related systems such as the EHR, perceived usefulness was significantly related to users' attitudes^11^. With that in mind, we hypothesised the following:

H4: Perceived usefulness positively influences MROs' and AMROs' attitudes toward the ICD-11.

#### Perceived ease of use

In general, individuals can devote only a limited amount of effort to several tasks that involve purview^51^. Therefore, the extent to which MROs or AMROs believe that using the ICD-11 is effortless is defined as perceived ease of use. Previous studies have shown an empirical relationship between perceived ease of use and attitude^52,53^. Despite the adequacy of resources and support, difficulty levels of healthcare technology are essential in determining users' attitudes^54^. In line with DTPB and other studies, the following hypothesis is proposed:

H5: Perceived ease of use positively influences MROs' and AMROs' attitudes toward ICD-11.

#### Compatibility

The compatibility construct defines the extent to which the MROs and AMROs opine that the ICD-11 fulfils their experience, values, and current needs. As agents of change, policymakers at the MOH must be aware of how technological innovations such as the ICD-11 fill the gap compared to previous versions of classification. To accurately ascertain users' needs, policymakers must have good relationships with users^55^. Subsequently, the new system introduced will be compatible with the users, which leads to a positive attitude among them^40^. This significant relationship was observed when using big data in disaster management^56^. Consistent with these previous studies, we propose the following:

H6: Compatibility positively influences MROs' and AMROs' attitudes toward ICD-11.

### Decomposition of subjective norm

#### Interpersonal influence

As the DTPB outlines, subjective norms are decomposed into interpersonal and external influences^12^. In the context of this study, the MROs and AMROs social group forms their interpersonal influence. Yeoh et al. found that social influence plays a significant role in behaviour^57^. Critical information, such as the use of the ICD-11, was shared among the users in this network. Previous studies have shown that interpersonal influence significantly influences users' subjective norms^58^. Therefore, the suggested hypothesis is as follows:

H7: Interpersonal influence positively influences MROs' and AMROs' subjective norms toward ICD-11.

#### External influence

Bhattacherjee defined external influence as influence from mass media, experts, and the government^59^. In this study's context, the external influence of ICD-11 use was not personal or unspecific to the MROs or AMROs. For any national-level implementation of systems introduced by the MOH, incentives provided by the government will be able to influence the subjective norm of the user^60^. Specifically, on the ICD-11 in Malaysia, the World Health Organization (WHO) actively engaged officers from the MOH. These findings subsequently led to efforts by the MOH to organise awareness sessions and prepare an e-learning platform to inform the MROs and AMROs of the advantages of the ICD-11. Therefore, we propose the following hypothesis:

H8: External influence positively influences MROs' and AMROs subjective norms toward ICD-11.

### Decomposition of perceived behavioural control

#### Self-efficacy

Perceived behavioural control is further decomposed into self-efficacy and facilitating conditions as defined in DTPB^47^. Under the framework of this research, self-efficacy is interpreted as MROs' and AMROs' confidence in using the ICD-11. If the respondents are confident, their choices, preparation, effort, mindset, and emotions will be geared toward the decision to use the ICD-11^41^. Previous studies focusing on the acceptance of Electronic Health Records (EHRs) among consumers and physicians have shown a positive significant relationship between self-efficacy and perceived behavioural control. If users are confident, they will opine that they have control over utilising a new system^11^. Therefore, we propose the following hypothesis:

H9: Self-efficacy positively influences MROs' and AMROs' perceived behavioural control toward ICD-11.

#### Facilitating conditions

Regarding DTPB, facilitating conditions are described as MROs' and AMROs' perceptions of the availability of resources to facilitate the use of the ICD-11^47^. In the use of the ICD-11, compared with the ICD-10, which is a manual process, the WHO has provided the ICD-11 Embedded Coding Tool (ICD-11 ECT) to assist in the search for suitable ICD-11 codes. The availability of this tool may affect users' perception of control in the use of ICD-11^6,61^. Concerning the acceptance of healthcare technologies, Mathai et al. showed that the availability of facilitating conditions improves consumers' perceived behavioural control ^62^. Hence, the following hypothesis is proposed:

H10: Facilitating conditions positively influence MROs' and AMROs' perceived behavioural control of ICD-11.

#### ICD-11 training

Finally, the ICD-11 training is defined as the cognitive activities involving MROs, AMROs, and trainers that result in knowledge transfer^63^. In ICD, training plays a central role in ensuring standardisation and the quality of the coded data^64^. This is especially important during the transition periods between the two versions of the ICD^8,26^. Previous studies on the relationship between training and facilitating conditions in a hospital setting have shown a positive significant relationship^36^. Therefore, we hypothesised the following:

H11: ICD-11 training positively influences the facilitating conditions of MROs and AMROs in the use of ICD-11.

## Method

A survey was conducted to collect data for this study using items adapted from previous studies^65^. The draft instrument was then translated and subjected to content validation before being disseminated for the pilot study^66^. The model was evaluated using PLS-SEM. Since the data distribution did not hinder the process, we assessed the research model for causality using a predictive technique.

### Instrument establishment

The items and scales used in this study's questionnaire were adapted from previous studies (Table 2). The choice of items adapted from previous studies was based on permission from the publisher and whether they have been used in the general healthcare contexts or have undergone validation^36,59,67–70^. Given that the referred literature was in English, the questionnaire for this study was first prepared in English. In Malaysia, Malay is the official language, whereas English is widely spoken and often used with Malay language on an interchangeable basis^71^. To enhance respondents' comprehension of the questions, we drafted the questionnaire in two languages instead of one in each language.

Hence, forward and backward translations between English and Malay were performed. For forward translation, two translators translated the instrument from English to Malay. The researchers and translators then harmonised the differences between the two translations. Then, two other translators uninvolved in the study translated the instrument backwards from Malay to English. Most of the items were identical to the original English, and any differences were discussed and finalised.

The questionnaire is made up of five parts. They are the information sheet with the consent form, Section A—demographic-related items, Section B—items related to intention to use ICD-11, Section C—items related to attitude, subjective norms, perceived behavioural control, perceived usefulness, perceived ease of use, self-efficacy, facilitating conditions, compatibility, interpersonal influence and external influence, and Section D—ICD-11 training and previous ICD coding experience. Sections B, C and D are the scale questions. A 7-point Likert scale, ranging from "strongly disagree" or "extremely unimportant" to "strongly agree" or "extremely important", was used to assess respondents' intentions and factors influencing it. We adopted the items in this section from earlier studies and adapted them to suit the research context and subjects. There were four to seven items for each variable, with 71 items identified for the initial draft of the questionnaire^36,69^.

Five public health and health informatics experts agreed to evaluate the scale (71 items) for its clarity, relevance, simplicity, and ambiguity^72^. The content validity for each of the domains was assessed using the following indicators: (1) the content validity index (I-CVI); (2) the scale-level content validity index based on average methods (S-CI/AVE); (3) the scale-level content validity index based on the universal agreement method (S-CVI/UA); (4) the probability of change agreement (P_c_); and; (5) the modified kappa (K) coefficient. We computed the CVI score with Microsoft Excel. After the expert panel's feedback, item FC6 was eliminated due to duplicity with FC3. It was also decided that some items were double-barrelled and subsequently restructured. Finally, the post-content validity instrument consisting of 78 items was verified.

### Pilot study

The post-content validity instrument was piloted and involved 105 participants. The pilot study ensures the instrument's feasibility before data collection^73^. The reliability of the data collected in the pilot study was tested using SPSS Statistics version 27.0 (IBM Corp., Armonk, NY, USA). All the domains were found to have Cronbach's alpha values of greater than 0.700. However, only 62 items with satisfactory factor loadings of more than 0.600 remained for the primary survey^74^.

### Data collection and preparation

All MROs and AMROs employed by the Ministry of Health Malaysia (MOH) composed the study population (N = 479). We used the power analysis tool G*Power 3.1 to determine the ideal sample size for the survey^75^. The tool indicated that a minimum of 85 respondents is needed. Referring to Memon et al., the suggested sample size of 160–300 valid observations with careful consideration of the target population was made for Structural Equation Modelling (SEM)^76^. For example, for a total population of 400, a sample size of 200 is considered large. Additionally, as DTPB is a complex model, a sample with fewer than 100 is not advised for multivariate statistical analysis techniques like CB-SEM and PLS-SEM^77^.

Respondents were chosen using the simple random sampling method. Similar to previous studies^78–80^, the chosen method was justified due to the homogeneity of tasks related to ICD use between the MROs and AMROs and ensuring the representativeness of the medical records professionals^10^. A serialised list of MROs and AMROs was obtained from the MOH. The prospective respondents were identified using a random number generator. We collected more than the minimum sample size required (n = 185) from the data collection exercise, which took four months. Then, the respondents were invited via email to complete the questionnaire via the attached Google Form link. Informed consent was obtained from all respondents. Respondents who agreed to participate provided consent online (by checking the "I agree" box) before answering the survey. No information on respondents' identifiers was collected, and respondents did not need to log into their accounts to access the Google Form. The Research Ethics Committee, Universiti Kebangsaan Malaysia (UKM PPI/111/8/JEP-2023-080) and the Medical Research & Ethics Committee, Ministry of Health (MOH) Malaysia (NMRR ID-23-00756-KIH (IIR) approved this study. The study was carried out following relevant guidelines and regulations.

### Data analysis

Using Microsoft Excel spreadsheet worksheet, data were tabulated and compared, especially to aid in ascertaining the model's predictive relevance. Data processing for descriptive statistics was done with SPSS Statistics version 27.0. For data normality, we used the WebPower online calculator to perform the normality test^81^. All 185 responses were included in the analysis; most respondents were females (n = 143, 77.3%). The respondents had a mean duration of experience with ICD of 8.4 years.

The data were quantitatively analysed using the structural equation modelling (SEM) approach. SEM allows researchers to estimate and model complex interactions between several independent and dependent variables at the same time. We used the partial least square SEM (PLS-SEM) because the structural model estimation involves a more complex model involving many constructs. Two assessment phases are recommended for PLS-SEM procedures: measurement and structural. To elucidate the correlations between variables, the path coefficient (β), t-value, p-value, coefficient of determination (R^2^), and effect size (f^2^) were evaluated^82^.

## Results

### Data preparation and descriptive statistics

We used the WebPower online tool for data outlier identification and normality. Table 1 shows that no problems related to outliers were detected^83,84^. Skewness and kurtosis values were computed to determine univariate normality^84^. There was no missing data detected. The mean, standard deviation, skewness, and kurtosis of each construct are displayed in Table 1. The kurtosis and skewness scores ranged from -0.730 to 0.984. Therefore, the data were normally distributed. Every construct yielded satisfactory mean scores. The lowest mean was for attitude (M = 2.095), and the highest mean was for self-efficacy (M = 4.242).

**Table 1:** Mean, standard deviation (SD), skewness and kurtosis.

Variable	Mean	SD	Skewness	Kurtosis
Intention to use ICD-11 (INT)	2.159	0.870	0.967	− 0.182
Attitude (ATT)	2.095	0.728	0.891	− 0.116
Subjective norm (SN)	2.293	1.036	0.982	0.253
Perceived behavioural control (PBC)	2.577	1.029	0.878	0.256
Perceived usefulness (PU)	2.961	1.668	0.687	− 0.282
Perceived ease of use (PEOU)	2.963	1.318	0.676	− 0.027
Compatibility (COM)	2.717	1.152	0.593	− 0.730
Interpersonal influence (II)	2.731	1.312	0.944	− 0.012
External influence (EI)	2.997	1.370	0.666	− 0.044
Self-efficacy (SE)	4.242	1.477	− 0.082	− 0.730
Facilitating conditions (FC)	2.554	0.839	0.902	0.817
ICD-11 training (TRA)	3.154	1.596	0.600	− 0.570
Previous ICD experience (EXP)	2.539	1.428	0.984	0.429

### Measurement model

**Figure 2:**
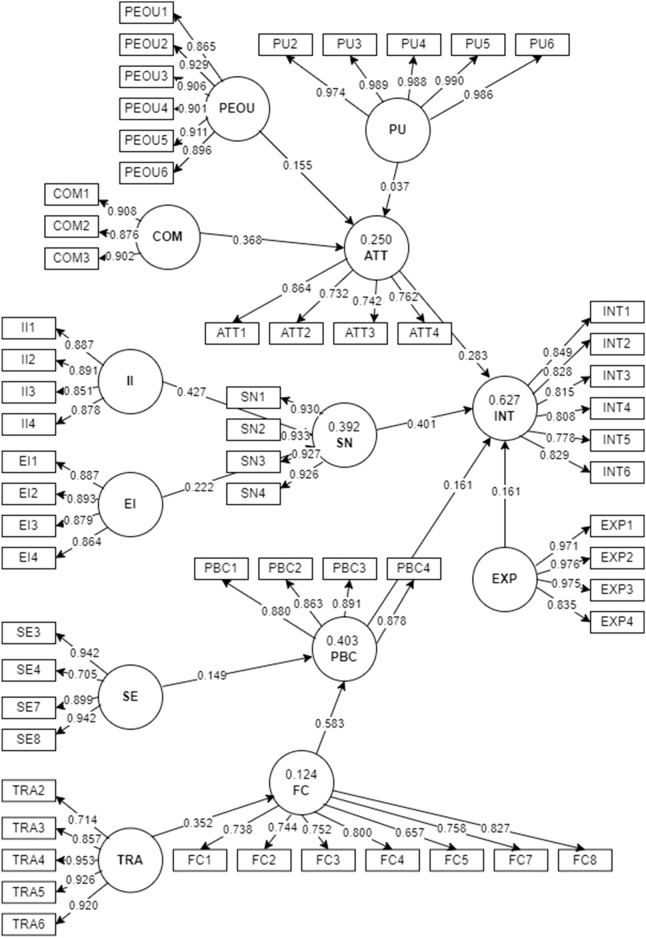
Measurement model.

We reported the validity and reliability of the model using the measurement model. The four phases of reflective measurement models are reflective indicator loadings, internal consistency reliability (ICR), convergent validity and discriminant validity^85^. Assessment of the reflective measurement model starts with examining the factor loadings. Factors with loadings higher than 0.500 were retained^86^. To compute the factor loadings of the items, we used SmartPLS 4.0.9.2^87^. Two items with loadings of < 0.500 (INT7; 0.485 and SE5; 0.476) were removed. This deletion process aimed to maintain the validity and reliability of the model^88^. The loadings (60 items) are summarised in Table 2 and Fig. 2. Self-efficacy (SE4; 0.705) had the lowest factor loading, while perceived usefulness had the highest factor loading (PU5; 0.990).

**Table 2:** Factor loading, reliability, and validity of the measurement model.

Construct	Reference	Items	Load	α	rho_A	CR	AVE
Intention to use ICD-11	Chang M-Y et al. ^36^ and Taylor & Todd ^68^	INT1	0.849	0.901	0.903	0.924	0.669
		INT2	0.828				
		INT3	0.815				
		INT4	0.808				
		INT5	0.778				
		INT6	0.829				
Attitude	Hung et al. ^69^ and Chang M-Y et al. ^36^	ATT1	0.864	0.780	0.798	0.858	0.603
		ATT2	0.732				
		ATT3	0.742				
		ATT4	0.762				
Subjective norm	Hung et al. ^69^ and Taylor & Todd ^68^	SN1	0.930	0.947	0.948	0.962	0.863
		SN2	0.933				
		SN3	0.927				
		SN4	0.926				
Perceived behavioural control	Hung et al. ^69^ and Taylor & Todd ^68^	PBC1	0.881	0.902	0.908	0.931	0.772
		PBC2	0.864				
		PBC3	0.891				
		PBC4	0.877				
Perceived usefulness	Davis ^89^	PU2	0.974	0.993	0.993	0.994	0.971
		PU3	0.989				
		PU4	0.988				
		PU5	0.990				
		PU6	0.986				
Perceived ease of use	Davis ^89^	PEOU1	0.865	0.954	0.966	0.963	0.813
		PEOU2	0.929				
		PEOU3	0.906				
		PEOU4	0.901				
		PEOU5	0.911				
		PEOU6	0.896				
Compatibility	Chau & Hu ^67^	COM1	0.908	0.877	0.883	0.924	0.802
		COM2	0.876				
		COM3	0.902				
Interpersonal influence	Hung et al. ^69^	II1	0.887	0.900	0.902	0.930	0.769
		II2	0.891				
		II3	0.851				
		II4	0.878				
External influence	Bhattacherjee ^59^	EI1	0.887	0.906	0.938	0.933	0.776
		EI2	0.893				
		EI3	0.879				
		EI4	0.864				
Self-efficacy	Hung et al. ^69^ and Taylor & Todd ^68^	SE3	0.942	0.865	0.935	0.903	0.661
		SE4	0.705				
		SE7	0.899				
		SE8	0.943				
Facilitating conditions	Taylor & Todd ^68^	FC1	0.725	0.887	0.890	0.910	0.560
		FC2	0.728				
		FC3	0.745				
		FC4	0.789				
		FC5	0.701				
		FC7	0.742				
		FC8	0.812				
ICD-11 training	Zaman et al. ^70^	TRA2	0.711	0.923	0.933	0.944	0.772
		TRA3	0.853				
		TRA4	0.955				
		TRA5	0.928				
		TRA6	0.922				
Previous ICD coding experience	Lee Y-H et al. ^90^	EXP1	0.971	0.956	0.964	0.969	0.886
		EXP2	0.976				
		EXP3	0.975				
		EXP4	0.835				

SE5 and INT7 were deleted due to low loadings.

### Common method variance

Since the independent and dependent variables in this study were collected simultaneously from the same respondent, common method variance (CMV) may have been an issue^91^. We adopted the single-common-method-factor approach to account for CMV^92^. A method factor was created using the PLS marker variable approach^93^. First, we chose seven items from the Social Desirability Scale that were gathered from the same survey but were not part of the model and were evaluated: (1) "I like to gossip at time;" (2) "There have been occasions where I took advantage of someone else;" (3) "I'm always willing to admit it when I made a mistake;" (4) "I sometimes try to get even rather than forgive and forget;" (5) "At times I have really insisted on having things my own way;" (6) "I have never been irked when people expressed ideas very different from my own;" (7) "I have never deliberately said something that hurt someone's feelings^94^." These items served as marker variables. Then, using the marker variable as an exogenous variable, a method factor was created where each endogenous construct was predicted. Finally, upon comparison with the baseline model, significant paths remain significant in the method factor model. Therefore, we conclude that there was no CMV issue with the data.

### Internal consistency reliability (ICR)

Using SmartPLS software, Rho_A, Cronbach's alpha and composite reliability (CR) were computed for the ICR^95^. Higher values for Rho_A suggest higher reliability levels. Values higher than 0.700 are considered satisfactory^86^. Cronbach's alpha and the CR are two additional metrics for assessing the reliability of internal consistency. The values of Cronbach's alpha must be higher than 0.700. Table 2 shows the CR, Cronbach's alpha and Rho_A values. The Rho_A, Cronbach's alpha, and CR values of all the constructs are promising, indicating a stable ICR.

### Convergent validity

Convergent validity is the degree to which a construct converges to represent the item variance. We used the average variance extraction (AVE) method to determine convergent validity. Each loading is squared with a minimum AVE of 0.500 (see Table 2). The "facilitating conditions" construct had the lowest AVE value (0.560). With an AVE of 0.971, the perceived usefulness construct achieves the maximum variance of (97.1%). The AVE values confirm the convergent validity of the model.

### Discriminant validity

Discriminant validity is the degree to which a variable differs from other variables empirically. The heterotrait-monotrait (HTMT) ratio was employed to examine discriminant validity^96^. Table 3’s HTMT values are less than 0.900, and its confidence intervals do not include a value of 1. Thus, we have demonstrated the discriminant validity of the study.

**Table 3:**
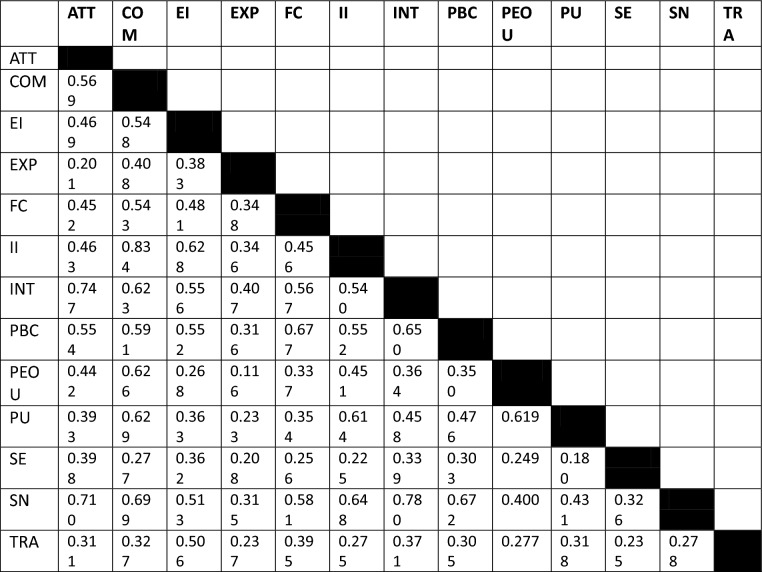
HTMT ratios.

### Structural model

We evaluated the multivariate skewness and kurtosis recommended by Hair et al. and Cain et al. ^97,98^. The findings demonstrated that the data collected were not multivariate normal. Mardia's multivariate skewness was (β = 38.815, p < 0.05), and Mardia's multivariate kurtosis was (β = 242.305, p < 0.05). As a result, we used a 5000-sample resample bootstrapping approach, as recommended by Hair et al., to provide the path coefficients, standard errors, t-values, and p-values for the structural model^99^.

**Figure 3:**
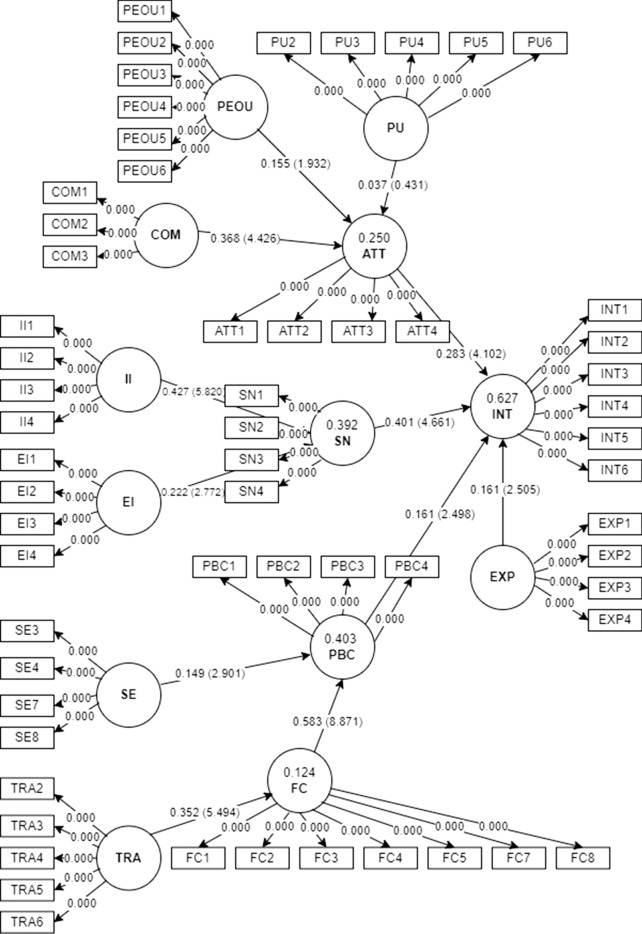
Structural model (t-value).

We assessed the structural model by bootstrapping the data involving 5000 subsamples, as summarised in Fig. 3. One hypothesis was deemed to be insignificant (H4). The other hypotheses (H1–H3, H5–H12) are significant at the 95% confidence interval, as shown in Table 4. Specifically, this study revealed that attitude (H1; β = 0.283; t = 4.102; p < 0.05), subjective norms (H2; β = 0.401; t = 4.664; p < 0.05), perceived behavioural control (H3; β = 0.160; t = 2.494; p = 0.006) and previous ICD coding experience (H12; β = 0.161; t = 2.506; p = 0.006) positively influence the intention to use ICD-11. On the predictors of attitude, we found that only perceived ease of use (H5; β = 0.155; t = 1.932; p = 0.027) and compatibility (H6; β = 0.368; t = 4.426; p < 0.05) positively affect attitude. On the other hand, perceived usefulness (H4; β = 0.037; t = 0.431; p = 0.333) does not significantly or positively influence attitude. For the predictors of subjective norm, this study showed that interpersonal influence (H7; β = 0.468; t = 5.820; p < 0.05) and external influence (H8; β = 0.174; t = 2.772; p = 0.003) positively affect the subjective norm of MROs and AMROs. Self-efficacy (H9; β = 0.150; t = 2.871; p = 0.002) and facilitating conditions (H10; β = 0.613; t = 8.832; p < 0.05) were linked to perceived behavioural control, and ICD-11 training (H11; β = 0.363; t = 5.504; p < 0.05) was linked to facilitating conditions.

**Table 4:** Significance tests and effect sizes (f^2^).

H	Path	B	t-value	p-values	Sig	BCI LL	BCI UL	F^2^
H1	Attitude → Intention to use ICD-11	0.283	4.102	< 0.05	Yes	0.168	0.394	0.130
H2	Subjective norm → Intention to use ICD-11	0.401	4.664	< 0.05	Yes	0.261	0.539	0.201
H3	Perceived behavioural control → Intention to use ICD-11	0.160	2.494	0.006	Yes	0.053	0.264	0.040
H4	Perceived usefulness → Attitude	0.037	0.431	0.333	No	− 0.101	0.179	0.001
H5	Perceived ease of use → Attitude	0.155	1.932	0.027	Yes	0.018	0.283	0.018
H6	Compatibility → Attitude	0.368	4.426	< 0.05	Yes	0.222	0.495	0.103
H7	Interpersonal influence → Subjective norm	0.468	5.820	< 0.05	Yes	0.334	0.599	0.244
H8	External influence → Subjective norm	0.174	2.772	0.003	Yes	0.091	0.353	0.054
H9	Self-efficacy → Perceived behavioural control	0.150	2.871	0.002	Yes	0.059	0.230	0.035
H10	Facilitating conditions → Perceived behavioural control	0.613	8.832	< 0.05	Yes	0.461	0.676	0.520
H11	ICD-11 training → Facilitating conditions	0.363	5.504	< 0.05	Yes	0.244	0.461	0.151
H12	Previous ICD coding experience → Intention to use ICD-11	0.161	2.506	0.006	Yes	0.055	0.267	0.062

For model fit evaluation, we used three indicators. They are the standardised root mean square residual (SRMR) and the exact fit criteria, d_ULS and d_G. The SRMR is the root mean square difference between the correlations that are observed and those implied by the model. Since SRMR is an absolute fit metric, zero denotes a perfect fit. A good fit is defined as a value less than 0.08^97^. The criteria d_ULS and d_G were additional reference points for fit evaluation. No cut-off values exist for the d_ULS or d_G indices^100^. Table 5 shows the acceptable model fit values of the index with 0.057 for SRMR, 6.396 for d_ULS and 3.609 for d_G.

**Table 5:** Model fit.

Model fit	
SRMR	0.057
d_ULS	6.396
d_G	3.609

Concerning the model's predictive relevance, the use of PLSpredict was proposed by Shmueli et al. ^88^. PLSpredict uses the holdout sample-based technique with a tenfold procedure to verify its predictive relevance and delivers case-level predictions on an item or construct level. According to Shmueli et al. ^88^, there is strong predictive power if all item differences (PLS-LM) are lower; if all are higher, predictive power is not confirmed; if the majority is lower, there is moderate predictive power; and if the minority is lower, there is low predictive power^88^. Table 6 shows that most of the PLS model errors were less than those of the LM model, indicating that the model proposed in this study has a moderate predictive ability.

**Table 6:** PLSpredict.

Item	PLS	LM	PLS-LM	Q^2^_predict
	RMSE	RMSE		
ATT1	0.898	0.998	− 0.100	0.200
ATT2	0.850	0.968	− 0.118	0.079
ATT3	0.900	1.103	− 0.203	0.116
ATT4	0.855	1.012	− 0.157	0.127
FC1	1.117	1.163	− 0.046	0.082
FC2	1.157	1.299	− 0.142	0.092
FC3	1.012	1.102	− 0.090	0.031
FC4	1.144	1.168	− 0.024	0.048
FC5	1.019	1.137	− 0.118	0.085
FC6	1.043	1.227	− 0.184	0.086
FC7	1.106	1.130	− 0.024	0.027
FC8	1.115	1.154	− 0.039	0.043
INT1	0.923	1.083	− 0.160	0.249
INT2	0.956	1.190	− 0.234	0.234
INT3	0.947	1.118	− 0.171	0.233
INT4	0.930	1.049	− 0.119	0.263
INT5	0.983	1.150	− 0.167	0.173
INT6	0.961	1.155	− 0.194	0.244
PBC1	1.150	1.152	− 0.002	0.052
PBC2	1.063	1.155	− 0.092	0.031
PBC3	1.205	1.170	0.035	0.069
PBC4	1.147	1.071	0.076	0.057
SN1	0.977	1.092	− 0.115	0.300
SN2	0.917	1.013	− 0.096	0.350
SN3	0.879	0.935	− 0.056	0.316
SN4	0.902	1.007	− 0.105	0.335

## Discussion

Several validity tests were performed to establish a valid and reliable model. From the literature, we adapted a total of 71 items. The instrument's content validity was assessed by five experts who scrutinised and scored the draft questionnaire. We then piloted the questionnaire among the study population and calculated the construct validity. As a result, eleven items were dropped, with 60 items used in the primary data collection.

Regarding internal consistency reliability, rho_A values between 0.700 and 0.950 are considered satisfactory. Higher values are undesirable as the items may be semantically redundant and may not be valid measures of the construct^86^. In the findings of our study, rho_A values for perceived usefulness, perceived ease of use and previous ICD coding experience were found to exceed 0.950. However, we opine that the high-reliability issue raised by Hair et al. ^97^ may not be the case for this study for three reasons. Firstly, the constructs are based on a sound theoretical foundation. In addition, items of the constructs were reviewed by experts based on its relevance and specific items in the constructs were reverse-coded to ensure respondents' attentiveness.

This study also established a model based on the DTPB to investigate the factors that could affect MROs' and AMROs' adoption of the ICD-11^12^. Factors influencing the intention to use ICD-11 include attitude, subjective norm, perceived behavioural control and previous ICD coding experience. Subjective norm was the strongest predictor of users' intention to use the ICD-11. Interpersonal and external factors significantly influence subjective norm, predicting the intention to use the ICD-11.

### Determinants of intention to use ICD-11

#### Subjective norm, interpersonal and external influences

In line with DTPB, the study has shown that subjective norm positively influences the intention to use the ICD-11^12^. Ultimately, MROs' and AMROs' decisions to use the ICD-11 depend on the opinions of people who are important to them and believe they should use the ICD-11. Of the factors considered, subjective norm had the most significant impact on MROs' and AMROs' intentions to use the ICD-11 (H2). This finding is inconsistent with other studies that found that factors such as attitude are stronger predictors^11,101,102^. This study also showed that interpersonal influence is significantly related to users' subjective norms (H7). Given that MROs and AMROs consist of a group of closely knit civil servants with a niche area of expertise in the healthcare sector, it is logical that social group relations influence the MROs and AMROs' intentions to use the ICD-11^10^.

This study also showed that external factors significantly influenced the subjective norm (H8). Our findings align with those of other studies that have shown that government and external agencies significantly influence the subjective norms of users^40,59^. In the context of the mandatory use of a system such as the ICD-11, the MOH has a role in ensuring the uniform adoption of the ICD-11 across the board. Mandating a behaviour such as using the ICD-11 will lead to the eventual adoption of the ICD-11. However, MOH must still allocate sufficient resources to win the hearts and minds of users and ensure that they are motivated to use the ICD-11. This is because the observed use is mainly due to users not having the choice but to use ICD-11, which could negatively impact the data quality^16^.

### Attitude, perceived usefulness, perceived ease of use and compatibility

Corresponding to DTPB^12^, attitude is another significant determinant influencing MROs' and AMROs' intention to utilise the ICD-11. Our study revealed that the users' favourable attitudes towards ICD-11 positively affect their intention (H1). Previous studies by Hsieh ^40^ and Mathai et al. ^11^ support the relationship between attitudes towards intentions to use newly introduced healthcare innovations. Moreover, our findings are also in accordance with the general attitudes among HIM professionals worldwide towards the ICD-11^103,104^.

Our study also confirmed that perceived ease of use (H5) and compatibility (H6) significantly influenced MROs' and AMROs' attitudes. If users perceive the ICD-11 as easy and fit well with their work, this will improve their tendency toward adopting the ICD-11. HIM professionals are known to operate in an increasingly complex and highly demanding environment^105^. A system that facilitates rather than adds to existing workloads is welcomed. Therefore, the MOH should actively engage the MROs and AMROs in system design and system use and in the subsequent implementation of suggestions to optimise the use of the ICD-11 at MOH facilities.

Contrary to the model and most related studies, we found that perceived usefulness is non-significant for attitude (H4)^11,69^. This means that the usefulness of the ICD-11 was not highly valued by respondents, indicating some ambivalence on its usefulness among MROs and AMROs. Moreover, Gajayanake et al. reported a complex relationship between how users perceive usefulness with attitude and their intention to use the innovation^106^. Nevertheless, at the time of data collection, the ICD-11 was still in the early phase of implementation, users were just being made aware of the new system, and most did not undergo formal training or use^14^. Future studies may investigate user perceptions after the ICD-11 was formally used at MOH facilities beginning in 2024.

### Perceived behavioural control, facilitating conditions, self-efficacy and ICD-11 training

Consistent with the DTPB^12^, this study showed that perceived behavioural control influences the intention to use the ICD-11 (H3). In other words, when MROs and AMROs feel that the existing systems and themselves are ready, they will intend to use the ICD-11. This outcome corroborates the findings of Hung et al. involving the use of an online system among healthcare workers^69^. In Malaysia, the MOH organised engagement sessions with all stakeholders before using the ICD-11 to clarify doubts and queries among stakeholders^107^. In addition, the system with the ICD-11 ECT uses the same interface as the previous system to give users a sense of familiarity and control and improve the intention to use the ICD-11 among MROs and AMROs^8,108^.

The study also proved that facilitating conditions and self-efficacy significantly influenced perceived behavioural control. If the MROs and AMROs are confident and resources are available at arm's length, they will most likely have a sense of control when using the ICD-11. This result aligns with studies involving new systems or innovations in healthcare settings, such as the ICD-11^69^. In addition, we found that ICD-11 training significantly influenced the facilitating conditions of MROs and AMROs toward the ICD-11. This finding is consistent with that of Aggelidis et al. in the context of healthcare facilities^109^. Overall, these factors give users a sense of empowerment for continued use of the ICD-11.

### Previous ICD coding experience

As proposed in the model, previous ICD coding experience significantly influenced the intention to use the ICD-11 (H12). That is, users with experience with the ICD-10 at MOH facilities were found to have favourable intentions to use the ICD-11. We opine that this could be due to the MOH's initiatives to identify the fears and uncertainty of MROs and AMROs. The MOH then took concrete steps to ensure the availability of learning materials^110^, coding guidelines^111^, engagement sessions^107^, and careful consideration to ensure minimal disruption of existing workflows^112^. Contrary to the findings of Alonso et al., in the context of ICD-9-CM and ICD-10-CM/PCS, it was reported that experienced users felt that it was more challenging to shift to a new system in comparison to users with minimal to no experience. This is because of the higher coding specificity required from the documented information^113^.

### Theoretical implications

The study is among the early studies examining factors influencing users' intention to use the ICD-11. Our work presents an empirically tested model for ICD-11 usage intention. The findings of our study significantly add to the body of knowledge already available on innovation intention-based models, particularly the intention to use ICD-11. First, we introduced an integrated innovation intention model based on DTPB. Two new variables—ICD-11 training and previous ICD coding experience have been incorporated into our suggested model.

Second, our proposed DTPB-based ICD-11 usage intention model offers a deeper comprehension of the users' psyche. Understanding human behaviour towards a newly introduced technology is valuable to the existing literature. The intention of users to adopt recently developed digital innovations can be measured and identified by our approach.

The final theoretical implication stems from the limitations of the current ICD-11 intention-based usage surveys. While previous research has documented the user experience, perceptions, and utilities, relatively few have investigated users' intentions to use ICD-11 and the factors influencing it ^8,26,114^. This limitation can be addressed by ensuring survey questions include information about factors affecting the intention to use ICD-11 among the users. Survey questions may cover topics such as perceptions towards ICD-11, social influences, and availability of resources. Our findings imply that users' subjective norm exhibits superior influence over all key constructs towards intention.

### Practical implications

The findings from this study will be especially beneficial to the government healthcare providers and key policymakers. Firstly, our study revealed a strong relationship between subjective norms and intention, suggesting that regular communication involving all levels and agencies within the MOH is important. When the right information is spread among the users, those in the know will inform the other users and relay the intended information. For example, information like ICD-11's usefulness, ease of use, and compatibility with current workflow. In addition, we have also found that experienced users who had previously used ICD have a more positive intention to use ICD-11. Therefore, we suggest that policymakers tap on the so-called "old timers" in the industry to act as influencers and get the rest of the users' buy-ins upon the commencement of the ICD-11 implementation.

The implementation planners also must concurrently ensure the availability of coding experts and infrastructure consistent with Ibrahim et al. ^8^. Moreover, it is logistically impossible for experts to be available physically and for the infrastructure to be the same at all health facilities. The policymakers at the MOH must ensure the basic infrastructure to use ICD-11 is available and that the experts are available via social messaging applications to answer the related queries^8,114^. As a result, users will feel empowered and more confident to use ICD-11.

From this study, we found that ICD-11 training significantly influences the facilitating conditions for the users. Therefore, as best as possible, the policymakers can get the necessary resources and plan training sessions for all users before ICD-11 implementation. This is because users will likely resist using ICD-11 if they are unaware of using ICD-11 properly. Therefore, the training sessions can also serve as an opportunity to clarify any worry and anxiety among the users brought about by implementing this initiative.

Our study findings will also guide policymakers in shaping their implementation strategies in Malaysia, involving similar new systems in the future for the anticipated adoption of the International Classification of Health Interventions (ICHI)^115–117^. At the MOH facilities, procedural coding is also done by the MROs and AMROs. Not only that, the characteristics of the application used for ICHI are similar to ICD-11. Therefore, this study's results could assist in preparing the implementation of related classification or terminology systems in the country (Supplementary Information).

## Limitations and future research

This study has several limitations. The first is the generalizability of the findings. This study only examined the perspective of MROs and AMROs from MOH in Malaysia. Depending on context and country, the process could involve other actors, such as physicians, administrative clerks, and policymakers, from codification to reporting results. Findings from studies conducted in multiple countries may provide more applicable and generalisable results. The respondents' recruiting technique may also have an impact on the results. The data were collected online. As a result, the sample may not accurately reflect the population of interest because it is a cohort of users accustomed to technology use. Future studies could extend these findings to examine changes in intentions postimplementation. In addition, the mixed methods methodology can incorporate rigorous qualitative data collection techniques to provide detailed information and improve the understanding of the factors impacting the intention to use the ICD-11.

## Conclusion

In conclusion, this study allows future studies to investigate better intentions to use the ICD-11 by utilising the instrument of this study. In addition, this study aimed to examine the essential factors influencing the intention to use the ICD-11 according to the DTPB. We also included previous ICD coding experience and ICD-11 training in the model. Both factors were found to be significant predictors of intention and facilitating conditions. The findings from this study may inform government policymakers from the users' perspective as health systems worldwide transition to ICD-11.

### Supplementary Information

Supplementary Information.

## Data Availability

The ethics approval guaranteed that the data would only be used for academic research purposes and that any sensitive or confidential information that could be used to identify or harm participants would be kept entirely confidential. Thus, the research data cannot be made public to preserve the participants' privacy. While this research is ongoing, the dataset is available upon reasonable request from the corresponding author.
