# Supplementary material for: Factors influencing the intention to use the ICD-11 among medical record officers (MROs) and assistant medical record officers (AMROs) in Ministry of Health, Malaysia
Source: Sci Rep. 2024 Apr 30;14:9926. doi: 10.1038/s41598-024-60439-2 (PMC11061162; doi:10.1038/s41598-024-60439-2)
Supplement: Supplementary file 1 — Supplementary Information. [file 41598_2024_60439_MOESM1_ESM.docx]

**LAMPIRAN A**

**BORANG SOAL SELIDIK**

**KAJIAN FAKTOR-FAKTOR YANG MEMPENGARUHI NIAT PENGGUNAAN ICD-11 DALAM KALANGAN PEGAWAI TADBIR DAN PENOLONG PEGAWAI TADBIR (REKOD PERUBATAN) DI FASILITI KEMENTERIAN KESIHATAN MALAYSIA**

Tujuan edaran soal selidik ini adalah untuk menilai faktor-faktor yang mempengaruhi niat penggunaan ICD-11 dalam kalangan Pegawai Tadbir dan Penolong Pegawai Tadbir (Rekod Perubatan) di Fasiliti KKM. Soal selidik ini mempunyai 4 Bahagian (A, B, C, D). Oleh itu, sukacita dipohon supaya Tuan/ Puan melengkapkan semua butiran seperti yang tertera. Semua maklumat adalah untuk kegunaan kajian ini sahaja.

| **BAHAGIAN A: MAKLUMAT SOSIODEMOGRAFI RESPONDEN**  ***SECTION A: RESPONDENT’S SOCIODEMOGRAPHY INFORMATION*** | | | |
| --- | --- | --- | --- |
| Sila lengkapkan butiran maklumat yang berkaitan di tempat yang disediakan atau sila tanda ‘/’ sebagai jawapan di kotak jawapan yang disediakan.  *(Please fill in the relevant information at the blank space or tick ‘/’ the relevant option in the box)* | | | |
| 1 | Jantina  *Sex* | Lelaki  *Male* |  |
|  |  | Perempuan  *Female* |  |
| 2 | Umur  *Age* |  | |
| 3 | Bangsa  *Race* | Melayu  *Malay* |  |
|  |  | Cina  *Chinese* |  |
|  |  | India  *Indian* |  |
|  |  | Others  *Lain-lain* |  |
| 4 | Tahap pendidikan  *Level of education* | Diploma/ STP/ STPM/ STAM/ HSC  *Diploma/ STP/STPM/ STAM/HSC* |  |
|  |  | Ijazah Sarjana Muda  *Bachelor’s degree* |  |
|  |  | Sarjana  *Masters* |  |
|  |  | Doktor Falsafah  *Doctorate* |  |
| 5 | Gred penjawatan  *Grade of appointment* | N44 |  |
|  |  | N41 |  |
|  |  | N40 |  |
|  |  | N36 |  |
|  |  | N32 |  |
|  |  | N29 |  |
|  |  | N26 |  |
|  |  | N22 |  |
| 6 | Hospital/ Fasiliti  *Hospital/ Facility* |  | |
| 7 | Bilangan tahun pengalaman pengekodan ICD  *Number of years of ICD coding* |  | |
| 8 | Adakah anda pernah bercuti >15 hari berturut-turut sejak setahun yang lalu?  *Have you taken any long uninterrupted leaves (>15 days) in the past one year?* | Ya  *Yes* |  |
|  |  | Tidak  *No* |  |

| **BAHAGIAN B: NIAT PENGGUNAAN ICD-11**  ***SECTION B: INTENTION TO USE ICD-11*** |
| --- |
| Sila baca setiap pertanyaan dengan teliti dan nyatakan pilihan anda dengan membulatkan angka yang **menggambarkan perasaan anda dengan paling tepat**  *Please read each descriptive statement carefully and indicate your choice by circling the appropriate number that is the number* ***that best describes how you feel about the statements.***   - Sangat Setuju/ *Strongly Agree* - Sangat Tidak Setuju/ *Strongly Disagree* |

| **Bil.** | **Niat penggunaan ICD-11 (INT)**  ***Intention to use ICD-11*** | **Sangat Sangat**  **Tidak Setuju Setuju** |
| --- | --- | --- |
| **Saya bersedia untuk…**  ***I am willing to…*** | | |
| INT1 | Meluangkan masa untuk mempelajari ilmu berkaitan ICD-11.  *Spend time learning about ICD-11 related knowledge.* | 1 2 3 4 5 6 7 |
| INT2 | Melibatkan diri dengan aktiviti berkaitan penggunaan ICD-11.  *Get involved in ICD-11 use related activities.* | 1 2 3 4 5 6 7 |
| INT3 | Meningkatkan kewibawaan kerjaya Rekod Perubatan dengan memanfaatkan pelbagai peluang demi memenuhi permintaan dalam penggunaan ICD-11.  *Improve the professional authority of the Medical Records profession by leveraging various opportunities to meet the demand for ICD-11 use.* | 1 2 3 4 5 6 7 |
| INT4 | Memanfaatkan pelbagai peluang untuk menimba pengetahuan bagi memenuhi permintaan kakitangan Rekod Perubatan yang berkemahiran dalam ICD-11.  *Take advantage of various opportunities to gain knowledge to meet the demand for ICD-11 specialised Medical Records personnel.* | 1 2 3 4 5 6 7 |
| INT5 | Memanfaatkan pelbagai peluang untuk menimba pengetahuan tentang cara penggunaan ICD-11 bagi memenuhi permintaan kakitangan Rekod Perubatan yang berkemahiran dalam ICD-11.  *Take advantage of various opportunities to gain knowledge on the application of ICD-11 to meet the demand for ICD-11 specialised Medical Records personnel.* | 1 2 3 4 5 6 7 |
| INT6 | Melibatkan diri secara aktif dalam kursus latihan berkaitan ICD-11 di tempat kerja.  *Actively attended on-the-job training courses on ICD-11.* | 1 2 3 4 5 6 7 |

| **BAHAGIAN C: SIKAP, NORMA SUBJEKTIF, TANGGAPAN KAWALAN TINGKAHLAKU PENGGUNAAN ICD-11, PERSEPSI KEBERGUNAAN, PERSEPSI MUDAH GUNA, EFIKASI KENDIRI, KEADAAN PEMUDAHCARA, KESERASIAN, FAKTOR DALAMAN & FAKTOR LUARAN**  ***SECTION C: ATTITUDE, SUBJECTIVE NORMS, PERCEIVED BEHAVIOURAL CONTROL, PERCEIVED USEFULNESS, PERCEIVED EASE OF USE, SELF-EFFICACY, FACILITATING CONDITIONS, COMPATIBILITY, INTERPERSONAL FACTOR & INTRAPERSONAL FACTOR*** |
| --- |
| Sila baca setiap pernyataan dengan teliti dan nyatakan pilihan anda dengan membulatkan angka yang **menggambarkan perasaan anda dengan paling tepat**.  *Please read each descriptive statement carefully and indicate your choice by circling the appropriate number that is the number* ***that best describes how you feel about the statements.***   - Sangat Setuju/ *Strongly Agree* - Sangat Tidak Setuju/ *Strongly Disagree* |

| **Bil.** | **Sikap (ATT)**  ***Attitude*** | **Sangat Sangat**  **Tidak Setuju Setuju** |
| --- | --- | --- |
| ATT1 | Pada pendapat saya, saya … dengan penggunaan ICD-11.  *In my opinion, I ... with the idea of coding in ICD-11.* | 1 2 3 4 5 6 7 |
| ATT2 | Penggunaan ICD-11 adalah menyenangkan.  *Using ICD-11 is pleasant.* | 1 2 3 4 5 6 7 |
| ATT3 | ICD-11 adalah berguna.  *It is useful to use ICD-11.* | 1 2 3 4 5 6 7 |
| ATT4 | Secara amnya, saya … dengan pengekodan diagnosis menurut ICD-11.  *In general, I … with the coding of diagnoses according to ICD-11.* | 1 2 3 4 5 6 7 |

| **Bil.** | **Norma subjektif (SN)**  ***Subjective norms*** | **Sangat Sangat**  **Tidak Setuju Setuju** |
| --- | --- | --- |
| **Kebanyakan orang yang penting kepada saya (rakan sekerja) berpendapat bahawa saya sepatutnya…**  ***Most people who are important to me (colleagues) would think that I should…*** | | |
| SN1 | Belajar tentang ICD-11.  *Learn about ICD-11.* | 1 2 3 4 5 6 7 |
| SN2 | Menggunakan ICD-11 di tempat kerja.  *Use ICD-11 at workplace.* | 1 2 3 4 5 6 7 |
| **Pihak (pegawai atasan) yang mempengaruhi keputusan saya akan berfikir bahawa saya sepatutnya…**  ***The people who influence (superiors) my decisions would think I should…*** | | |
| SN3 | Mempelajari ICD-11.  *Learn ICD-11.* | 1 2 3 4 5 6 7 |
| SN4 | Menggunakan ICD-11 di tempat kerja.  *Use ICD-11 at workplace.* | 1 2 3 4 5 6 7 |

| **Bil.** | **Tanggapan kawalan tingkah laku (PBC)**  ***Perceived behavioural control*** | **Sangat Sangat**  **Tidak Setuju Setuju** |
| --- | --- | --- |
| **Saya mempunyai sumber, pengetahuan dan kemampuan untuk…**  ***I have the resources, knowledge and ability to…*** | | |
| PBC1 | Belajar tentang ICD-11.  *Learn about ICD-11.* | 1 2 3 4 5 6 7 |
| PBC2 | Menggunakan ICD-11.  *Use ICD-11.* | 1 2 3 4 5 6 7 |
| **Saya mampu untuk…**  ***I would be able to…*** | | |
| PBC3 | Mempelajari kaedah penggunaan ICD-11.  *Learn the methods of using ICD-11.* | 1 2 3 4 5 6 7 |
| PBC4 | Mengamalkan kaedah penggunaan ICD-11.  *Apply the methods of using ICD-11.* | 1 2 3 4 5 6 7 |

| **Bil.** | **Persepsi kebergunaan (PU)**  ***Perceived usefulness*** | **Sangat Sangat Tidak**  **Setuju Setuju** |
| --- | --- | --- |
| **Penggunaan ICD-11…**  ***Using ICD-11…*** | | |
| PU2 | Dalam pekerjaan saya akan membantu saya menyelesaikan tugasan dengan lebih cepat.  *In my work will help me to complete tasks faster.* | 1 2 3 4 5 6 7 |
| PU3 | Akan meningkatkan prestasi pekerjaan saya.  *Will improve my work performance.* | 1 2 3 4 5 6 7 |
| PU4 | Dalam pekerjaan saya akan meningkatkan produktiviti saya.  *In my work will increase my productivity.* | 1 2 3 4 5 6 7 |
| PU5 | Akan meningkatkan keberkesanan pekerjaan saya.  *Will increase my work effectiveness.* | 1 2 3 4 5 6 7 |
| PU6 | Memudahkan pekerjaan saya.  *Makes my work easy.* | 1 2 3 4 5 6 7 |

| **Bil.** | **Persepsi mudah guna (PEOU)**  ***Perceived ease of use*** | **Sangat Sangat Tidak Setuju Setuju** |
| --- | --- | --- |
| PEOU1 | Bahan rujukan berkaitan dengan ICD-11 jelas dan mudah difahami.    *Reference materials on ICD-11 are clear and easy to understand.* | 1 2 3 4 5 6 7 |
| **Saya dapat melakukan perkara berikut dengan mudah…**  ***I will find it easy to…*** | | |
| PEOU2 | Belajar untuk menggunakan ICD-11.  *Learn to use ICD-11.* | 1 2 3 4 5 6 7 |
| PEOU3 | Mencari kod ICD-11 yang tepat.  *Find the correct ICD-11 codes.* | 1 2 3 4 5 6 7 |
| PEOU4 | Menjadi mahir menggunakan ICD-11.  *Become proficient using ICD-11.* | 1 2 3 4 5 6 7 |
| **Saya mendapati bahawa ICD-11…**  ***I find that ICD-11 is…*** | | |
| PEOU5 | Fleksibel dari segi penggunaan.  *Flexible in terms of use.* | 1 2 3 4 5 6 7 |
| PEOU6 | Mudah untuk digunakan.  *Easy to use.* | 1 2 3 4 5 6 7 |

| **Bil.** | **Keserasian (COM)**  ***Compatibility*** | **Sangat Sangat Tidak**  **Setuju Setuju** |
| --- | --- | --- |
| **Penggunaan ICD-11…**  ***Using ICD-11…*** | | |
| COM1 | Sesuai dengan cara saya bekerja.  *Is compatible with the way I work.* | 1 2 3 4 5 6 7 |
| COM2 | Serasi dengan amalan pekerjaan saya.  *Is compatible with my working practices.* | 1 2 3 4 5 6 7 |
| COM3 | Memenuhi keperluan pekerjaan saya.  *Meets the needs of my work.* | 1 2 3 4 5 6 7 |

| **Bil.** | **Faktor dalaman (II)**  ***Interpersonal influence*** | **Sangat Sangat Tidak**  **Setuju Setuju** |
| --- | --- | --- |
| II1 | Rakan-rakan saya berfikir bahawa saya harus menggunakan ICD-11.  *My friends think that I should use ICD-11.* | 1 2 3 4 5 6 7 |
| II2 | Rakan-rakan sejawat saya berfikir bahawa saya harus menggunakan ICD-11.  *My colleagues think that I should use ICD-11.* | 1 2 3 4 5 6 7 |
| **Kenalan saya…**  ***My acquaintances…*** | | |
| II3 | Beranggapan bahawa penggunaan ICD-11 ialah idea yang bagus.  *Consider that the use of ICD-11 is a good idea.* | 1 2 3 4 5 6 7 |
| II4 | Mempengaruhi saya untuk mencuba menggunakan ICD-11.  *Have influenced me to try using ICD-11 in clinical coding.* | 1 2 3 4 5 6 7 |

| **Bil.** | **Kebolehinginan sosial (SD)**  ***Social desirability scale*** | **Sangat Sangat Tidak**  **Setuju Setuju** |
| --- | --- | --- |
| SD1 | Ada kalanya saya suka bergosip.  *I like to gossip at times.* | 1 2 3 4 5 6 7 |
| SD2 | Ada masanya saya mengambil kesempatan terhadap orang lain.  *There have been occasions where I took advantage of someone else.* | 1 2 3 4 5 6 7 |
| SD3 | Saya selalunya sanggup mengaku apabila saya melakukan kesalahan.  *I’m always willing to admit it when I made a mistake.* | 1 2 3 4 5 6 7 |
| SD4 | Kadang-kadang saya cuba membalas dendam daripada memaafkan dan melupakannya.  *I sometimes try to get even rather than forgive and forget.* | 1 2 3 4 5 6 7 |
| SD5 | Ada kalanya saya berkeras untuk melakukan sesuatu mengikut cara saya.  *At times I have really insisted on having things my own way.* | 1 2 3 4 5 6 7 |
| SD6 | Saya tidak pernah merasa jengkel apabila orang lain mempunyai idea yang berbeza dengan saya.  *I have never been irked when people expressed ideas very different from my own.* | 1 2 3 4 5 6 7 |
| SD7 | Saya tidak pernah dengan sengaja mengeluarkan kata-kata yang menggores hati orang lain.  *I have never deliberately said something that hurt someone’s feeling.* | 1 2 3 4 5 6 7 |

| **Bil.** | **Faktor luaran (EI)**  ***External influence*** | **Sangat Sangat Tidak**  **Setuju Setuju** |
| --- | --- | --- |
| EI1 | Saya pernah membaca terbitan KKM bahawa penggunaan ICD-11 ialah cara yang bagus untuk pengekodan klinikal.  *I have read MOH publications which stated that using ICD-11 is a good way to perform clinical coding.* | 1 2 3 4 5 6 7 |
| EI2 | Saya pernah membaca terbitan WHO yang mengatakan bahawa penggunaan ICD-11 ialah cara yang bagus untuk melakukan pengekodan klinikal.    *I have read WHO publications which stated that using ICD-11 is a good way to perform clinical coding.* | 1 2 3 4 5 6 7 |
| **KKM dan/atau WHO…**  **MoH and/or WHO…** | | |
| EI3 | Berusaha untuk memberikan sentimen positif berkenaan ICD-11.  *Depicted a positive sentiment for using ICD-11.* | 1 2 3 4 5 6 7 |
| EI4 | Menerbitkan bahan bacaan yang mempengaruhi saya untuk mencuba pengekodan ICD-11.  *Published materials that influenced me to try ICD-11 coding.* | 1 2 3 4 5 6 7 |

| **Bil.** | **Efikasi kendiri (SE)**  ***Self-efficacy*** | **Sangat Sangat Tidak**  **Setuju Setuju** |
| --- | --- | --- |
| **Saya…**  ***I…*** | | |
| SE3 | Selesa menggunakan ICD-11 dengan sendiri.  *Will feel comfortable to use ICD-11 by myself.* | 1 2 3 4 5 6 7 |
| SE4 | Kemampuan untuk menggunakan ICD-11 dengan sendiri tanpa rasa kekok adalah…  *The ability to independently code with ICD-11 comfortably is …* | 1 2 3 4 5 6 7 |
|  | | **Sangat Sangat Tidak**  **Penting Penting** |
| SE7 | Adalah … untuk saya berasa selesa mengekod dalam ICD-11 tanpa bantuan.  *Being comfortable coding ICD-11 on my own is…* | 1 2 3 4 5 6 7 |
| SE8 | Adalah ... untuk tiada sesiapa yang dapat membimbing saya dalam pengekodan ICD-11.  *Being able to code in ICD-11 even if no one is around to tell me how to use it is...* | 1 2 3 4 5 6 7 |

| **Bil.** | **Keadaan pemudahcara (FC)**  ***Facilitating conditions*** | **Sangat Sangat Tidak**  **Setuju Setuju** |
| --- | --- | --- |
| **Saya…**  ***I…*** | | |
| FC1 | Boleh menggunakan ICD-11 pada bila-bila masa saya mahu menggunakannya.  *Can use ICD-11 at any time that I want to.* | 1 2 3 4 5 6 7 |
| FC2 | Memiliki kemahiran pengekodan terdahulu yang boleh membantu saya dengan pengekodan ICD-11.  *Have prior coding skills that can help with ICD-11 coding.* | 1 2 3 4 5 6 7 |
| FC3 | Berusaha untuk menggunakan ICD-11.  *Make an effort to use ICD-11.* | 1 2 3 4 5 6 7 |
| FC4 | Mempunyai masa yang mencukupi untuk membiasakan diri dengan ICD-11.  *Have enough time needed to familiarise myself with using ICD-11.* | 1 2 3 4 5 6 7 |
| FC5 | Mempunyai masa yang mencukupi untuk menggunakan ICD-11.  *Have sufficient time to code in ICD-11.* | 1 2 3 4 5 6 7 |
| FC7 | Mempunyai akses kepada sumber (komputer) yang diperlukan untuk menggunakan ICD-11.  *Have access to necessary resources (computer) required to use ICD-11.* | 1 2 3 4 5 6 7 |
| FC8 | Mempunyai akses kepada sumber (internet) yang diperlukan untuk menggunakan ICD-11.  *Have access to necessary resources (internet) required to use ICD-11.* | 1 2 3 4 5 6 7 |

| **BAHAGIAN D: LATIHAN ICD-11 & PENGALAMAN PENGEKODAN VERSI ICD TERDAHULU**  ***SECTION D: ICD-11 TRAINING & PREVIOUS CODING EXPERIENCE*** |
| --- |
| Sila fahami pernyataan di bawah dan **nyatakan pilihan yang paling tepat** dengan membulatkan nombor yang sesuai berdasarkan skala berikut.  *(Please read each descriptive statement carefully and indicate your choice by circling the appropriate number that is the number* ***that best describes how you feel about the statements****)* |

| **Bil.** | **Latihan ICD-11 (TRA)**  ***ICD-11 training*** | **Sangat Sangat Tidak**  **Setuju Setuju** |
| --- | --- | --- |
| TRA2 | Ada individu (atau kumpulan) khusus yang bersedia untuk membantu saya dengan pengekodan ICD-11 jika perlu.  *A specific person (or group) is available when needed for assistance with ICD-11 coding.* | 1 2 3 4 5 6 7 |
| TRA3 | Arahan khusus berkenaan dengan ICD-11 ada di tempat kerja saya.  *Specialized instructions concerning ICD-11 are available to me at workplace.* | 1 2 3 4 5 6 7 |
| TRA4 | Latihan yang khas berkenaan ICD-11 disediakan di tempat kerja.  *Specialized education concerning ICD-11 are available to me at workplace.* | 1 2 3 4 5 6 7 |
| TRA5 | Program khas berkenaan latihan dalam ICD-11 disediakan di tempat kerja.  *Specialized programs about training on the ICD-11 are available to me at workplace.* | 1 2 3 4 5 6 7 |
| TRA6 | Perunding khas berkenaan latihan dalam ICD-11 disediakan di tempat kerja.  *Specialized consultants about training on the ICD-11 are available to me at workplace.* | 1 2 3 4 5 6 7 |

| **Bil.** | **Pengalaman pengekodan versi ICD terdahulu (EXP)**  ***Previous ICD coding experience*** | **Sangat Sangat Tidak**  **Setuju Setuju** |
| --- | --- | --- |
| EXP1 | Saya suka menggunakan ICD-10.  *I enjoy using ICD-10.* | 1 2 3 4 5 6 7 |
| EXP2 | Saya selesa mengekod dalam ICD-10.  *I am comfortable coding using ICD-10.* | 1 2 3 4 5 6 7 |
| EXP3 | Saya selesa untuk mencari kod ICD-10 yang sesuai.  *I am comfortable searching for ICD-10 codes.* | 1 2 3 4 5 6 7 |
| EXP4 | Saya boleh menghafal kod ICD-10 yang kerap digunakan di tempat kerja saya.  *I can memorize common ICD-10 codes in my facility.* | 1 2 3 4 5 6 7 |
